# Regulatory assessment of nano-enabled health products in public health interest. Position of the scientific advisory board of the French National Agency for the Safety of Medicines and Health Products

**DOI:** 10.3389/fpubh.2023.1125577

**Published:** 2023-03-02

**Authors:** Wahiba Oualikene-Gonin, Valérie Sautou, Eric Ezan, Henri Bastos, Eric Bellissant, Laëtitia Belgodère, Patrick Maison, Joël Ankri, The Scientific Advisory Board of the ANSM, Joël Ankri

**Affiliations:** ^1^Agence nationale de sécurité du médicament et des produits de santé, Saint-Denis, France; ^2^Université Clermont Auvergne, CHU Clermont Ferrand, Clermont Auvergne INP, CNRS, ICCF, Clermont-Ferrand, France; ^3^Commissariat à l'énergie atomique et aux énergies alternatives, Institut national de recherche pour l'agriculture, l'alimentation et l'environnement, Département Médicaments et Technologies pour la Santé (DMTS), Université Paris-Saclay, Gif-sur-Yvette, France; ^4^Agence nationale de sécurité sanitaire de l'alimentation, de l'environnement et du travail, Maisons-Alfort, France; ^5^Service de Pharmacologie, Centre Régional de Pharmacovigilance, de pharmaco-épidémiologie et d'information sur le médicament, CHU de Rennes, UMR INSERM 1085 - Institut de Recherche en Santé, Environnement et Travail (IRSET), Université de Rennes 1, Rennes, France; ^6^EA 7379, Faculté de Santé, Université Paris-Est Créteil, Créteil, France; ^7^CHI Créteil, Créteil, France; ^8^Université de Versailles St Quentin- Paris Saclay, Inserm U1018, Versailles, France

**Keywords:** nanomaterials, public health, regulatory science, nanomedicines, innovative drugs, medical devices, medicines, excipients

## Abstract

Nanomaterials are present in a wide variety of health products, drugs and medical devices and their use is constantly increasing, varying in terms of diversity and quantity. The topic is vast because it covers nanodrugs, but also excipients (that includes varying proportions of NMs) and medical devices (with intended or not-intended (by-products of wear) nanoparticles). Although researchers in the field of nanomedicines in clinical research and industry push for clearer definitions and relevant regulations, the endeavor is challenging due to the enormous diversity of NMs in use and their specific properties. In addition, regulatory hurdles and discrepancies are often cited as obstacles to the clinical development of these innovative products. The scientific council of the Agence Nationale de Sécurité du Médicament et des produits de santé (ANSM) undertook a multidisciplinary analysis encompassing fundamental, environmental and societal dimensions with the aim of identifying topics of interest for regulatory assessment and surveillance. This analysis allowed for proposing some recommendations for approximation and harmonization of international regulatory practices for the assessment of the risk/benefit balance of these products, considering as well the public expectations as regards efficacy and safety of nanomaterials used in Health products, in terms of human and environmental health.

## Introduction

A wide variety of manufactured nanomaterials (NMs) are present in a considerable number of health products (1, 2). Because of their nanometric size, they have particular properties that are exploited for maximizing efficacy and reducing dose and toxicity. This can be done along different lines such as drug targeting, controlled and site-specific release, preferential distribution in the body (e.g., in areas with cancerous lesions), or improved transport across biological barriers (1). They may also have specific risks and toxicity (2). As complex and innovative products, they pose public health challenges to the whole society and to regulatory agencies in charge of clinical trial or marketing authorizations (3, 4). Indeed, in addition to nanomedicine products, nanotechnology may be involved in medical devices (MDs), and conventional excipients. Because of the impact on regulatory assessment and surveillance, and its public health importance, the scientific board of ANSM decided to tackle the topic of nanotechnologies in health products from fundamental to environmental and societal dimensions.

In general, for medicines, the main questions are those related to pharmaceutical quality, efficacy and non-clinical and clinical safety, in order to establish the risk-benefit balance for marketing authorization. Subsequently, it is necessary to use relevant vigilance tools for a continuous risk-benefit assessment approach. These steps should be adapted to all health products enabled with nanotechnology also because NMs are a concern to the general public (5). The use of NMs in many fields (clothing, food, cosmetics), has undeniably led to questions from the public about these technologies (6). The regulatory landscape is sketchy with very few guidance and consensus definitions (7), although a new guidance was recently issued by the Food and Drug Administration (FDA) (8).

Outlining the main challenging points and unknowns for regulatory assessment and surveillance, taking also into account societal and environmental questions in the larger context of public health, recommendations are proposed for approximation or harmonization of regulatory approaches, considering all the items that are to be assessed before and after marketing authorization. Some scientific gaps were also identified, which could help define relevant and useful assays and methods to achieve streamlined and straightforward regulatory assessment processes. This is an initiative in line with recent efforts to level translational gaps through contact with regulators and regulatory science development (9).

## Challenging points in regulatory assessment and surveillance

According to the recommendation of the European Commission (10), the definition of a nanomaterial is generally applied for excipients and MDs, while nanomedicines have active ingredients or part of the active ingredient (e.g., vector) and a more specific definition. The European Medicines Agency (EMA) criteria for a nanoparticle used in a drug are systems manufactured for clinical applications, having at least one component in the nanoscale and having specific properties giving them a clinical advantage related to nanoengineering and size (7).

We can therefore distinguish:

- Medicines designed deliberately at the nanoscale called “nanodrugs” or “nanomedicines” with the aim of crossing physiological barriers and bringing active substances more quickly and/or more precisely into the body), which may also help reduce toxicity. Products concerned are mainly nanomedicines and some MDs.
- Medicinal products containing NMs without direct therapeutic purposes with multiple uses (such as opacifier, dye, UV protection, etc.). These include for instance, conventional excipients (that may harbor a proportion of NMs) and by-products of MDs wear.

### Pharmaceutical quality of nanodrugs

At the moment, quality assessments are made on a case-by-case basis (11), allowing authorities to require any additional data that may be needed to perform a complete and thorough assessment (3). Quality-by-design approaches are also being encouraged and developed for NMs used in health products (4, 12, 13).

Knowledge is not yet exhaustive, limitations exist in current methods of analysis, control and metrology (14).

Overall, important points of attention in relation to the pharmaceutical quality of nanoparticle size compounds are to be highlighted:

- Development, identification, and standardization of relevant and adapted characterization methods. The development and standardization of missing methods has been taken into account by the European Commission Joint Research Center REFINE project (14) and aims to benefit from the joint efforts of method developers and standardization communities.
- Definition of regulatory standards on expected characterizations and critical quality attributes as well as critical process parameters are required [quality-by-design (4, 15)].

### Non-clinical studies of nanodrugs

The “conventional” toxicological approach proposed by the current guidelines for drugs in general (International Council for Harmonization of Technical Requirements for Pharmaceuticals for Human Use (ICH), FDA, EMA) is applicable. Nevertheless, in general, existing methods may be improved:

- Regarding genotoxicity, the *in vitro* Ames test assay, which did provide false negatives after challenge with NMs (sometimes NMs simply does not enter the bacterium and therefore has no effect) has been considered not relevant for assessing mutagenicity (16). The Nanogenotox project (17) has made it possible to point out the difficulty of obtaining the same results between different laboratories, on the characterization of NMs sizes, but also on the effects observed.
- Carcinogenicity studies, which are only mandatory if the drug is given in the long term, are long studies (about 2 years). For some NMs (e.g., nickel NMs), carcinogenicity mechanisms are not fully understood and specific methods must be defined and validated (18).
- For immunotoxicity, it is known that NMs can interact with the immune system leading to immune reactions with an impact on the efficacy and/or safety of the medicinal product (19). Indeed, cases of CARPA syndrome (a pseudo-allergy syndrome related to complement C-activation) which have occurred with several NMs systems, have been reported (20). At present, there is no specific regulatory framework (15, 21). New models, eventually leading to new regulatory standards, need to be developed (15).

It is also noteworthy that the EU Horizon 2020 project REFINE has recently developed a Decision Support System (DSS) to help stakeholders with relevant testing strategies for efficient preclinical assessment of nanotechnology-enabled products. This DSS helps developers and regulators to prioritize assays to be performed, especially regarding immunotoxicological endpoints for efficient preclinical assessment of nano-enabled products (22).

### Nanosimilars

Made in the same spirit as generic drugs, they can be considered as generic nanomedicines (14). Comparability reference documents for generic drugs have been written in a multidisciplinary manner with quality, non-clinical and clinical experts. In the case of similar NMs, and this is different from what is done for generics, it must be demonstrated that pharmacokinetics (*in vivo* models) and elimination are similar.

Differences in liposome characteristics may not be detectable by conventional bioequivalence tests alone and that further studies are needed (23). The European Commission's Joint Research Center recommends drawing inspiration from what has been done for biosimilars to develop a generic nanodrug regulatory approach (14).

In nanosimilars, every difference, even a small one, in manufacturing can have an impact on these properties, especially pharmacokinetics and pharmacodistribution such as for Doxil^®^ and Lipodox^®^ which are liposomal solutions containing the same qualitative and quantitative compositions. (24–26). The actions to be taken could be of developing new models that are more relevant in terms of pharmacokinetics and pharmacodistribution and in general models for determining the equivalence of nanosimilars. Then the other component would concern the evolution of regulatory approaches according to these new models, drawing on what has been done for biosimilars (27).

### Excipients

Some food additives at the nanoscale size, authorized by European Food Safety Authority (EFSA), can be present in marketed European medicinal drug products up to 50% in concentration (28).

Currently, the main topic in the field of nanoparticle excipients is that titanium dioxide used as food additive (E171) is widely used in drugs and cosmetics, and has recently been banned from use in food in Europe (28). In the EU, the question hence arises of its maintenance in health products.

In Europe, around 8,000 pharmaceutical marketed drugs are concerned frequently for oral solid forms (29).

It should be noted that discussions are also ongoing for other dyes such as E172 [red, iron-based (30)], and silicates [Aerosil^®^ (31)] which contain nanoparticle forms.

The interest of the nanoscale excipients used in various drugs in terms of stability, resistance to light but also in terms of patient compliance (color and physical aspect of the administered forms) have to be analyzed considering potential risks and doubt arising from the nanoscale.

### Clinical assessment

The benefit risk balance assessment for these products is similar to others with clinical trials and vigilance activities. Adverse effects related to nanodrugs such as pseudo-allergies (or CARPA syndromes) and immunotoxicological effects are the main that have been previously reported with nanomedicines (2, 20). Comparison of side-effects of drugs formulated with nanoparticle or not, showed induction of pseudo-allergic responses associated with NMs. As different compounds were involved, size is undoubtedly a factor (19). More research is needed to determine whether some specific classes of compounds are involved. Potential risks are therefore essentially related to immunotoxicological effects (2). These potential risks are therefore to be considered when establishing the benefit risk balance.

### Medical devices (MDs)

MDs products may intentionally and intrinsically contain NMs or they can release (by wear or other mechanism) particles, some of which may be nanoparticular in size.

Indeed, NMs can be added for different reasons, for example to confer new properties (surface coatings, imaging), but can also be the unintended consequences of wear that can lead to particle releases, some of which are at the nanoscale (32, 33).

Since the recent entry into force of the Regulation on MDs (Regulation (EU) 2017/745) (34), NMs in MDs are now regulated, and this regulation uses the classic definition of a nanomaterial from the 2022 EC Recommendation (10).

The European Scientific Committee on Emerging and Newly Identified Health Risks (SCENIHR) has also published a recommendation on the use of NMs in MDs, including information on risk assessments specifically for these NMs (35).

From the new regulation and the conclusions of SCENIHR, it can be outlined:

- That no provision on the identification of NMs composing MDs is laid down in the Regulation. As the regulation currently stands, it is not possible to identify MDs containing NMs. This has implications for MDs surveillance (materiovigilance and surveillance epidemiology) and transparency.
- Evaluation and models do not consider the aging of MDs (even if this point is included in the ISO 10993-22 standard). The models used to assess the release of nanoparticles from MDs do not sufficiently consider the aging of MDs (even if the characterization of nanoparticles is well-described in the ISO 10993-22 standard). Indeed, existing models do not sufficiently take into account the exact MD implantation site with its environment and its duration of implantation in the organism. A case-by-case approach is still needed for the risk assessment of MDs as no currently available test method has been validated specifically for NMs. The application of such tests will have to be done on a case-by-case basis with a particular adaptation to the specificities of NMs according to the use for which they are intended.

### Surveillance of health products

After marketing, health products containing NMs, like any other, are subject to constant monitoring. Adverse drug reporting may be done by health professionals or patients as any other product and allow to detect signal. In the case of detection of a signal, retrospective epidemiological studies may allow to confirm this signal or not. All this surveillance is based on the product and each of its components (such as excipients).

Therefore, epidemiological studies are important tools for the surveillance of health products. However, for the surveillance of product with nanoparticles, for instance as an excipient, it is necessary to know all products containing nanoparticles and their characteristics.

For MDs, they may intentionally contain intentional NMs or release them due to wear and tear. In both cases, in order to ensure materiovigilance, it would be desirable to ensure traceability. In the case of unintentional release, it is more specifically a question of also stimulating specific research, or even the development of relevant models to predict the consequences of these releases in the long term. This would also be useful to initiate surveillance studies on the consequences of unintentional releases of NMs by certain MDs.

Pharmacovigilance as it exists makes it possible to detect consequences and epidemiological studies could confirm and quantify especially for products deployed on large population scales.

Common adverse effects related to NMs and not to the active substance may be identify.

Therefore, it would be essential to identify products with the presence (nature and quantity) of each type of NMs to ensure health products containing NMs surveillance.

## Societal topics

The novelty of the object, the supposed unpredictability of its reactions and interactions in its changes in form and properties, combined with the uncertainty that affects knowledge about its medium- and long-term effects on living organisms and the environment, maintain public mistrust and the awareness of scientific communities (6).

The debates surrounding the use of NMs in medicine are part of a broader framework, that of the controversies over nanotechnologies (36). It seems important to assess acceptability and to implement measures to raise awareness of the applications, uses and benefits of these technologies (37).

The European Chemical Agency (ECHA) carried out a study in 2020 on the public perception of NMs and their safety in the EU (38). Although awareness of the presence of NMs in marketed products increased between 2005 and 2020 (from 43 to 65% of those surveyed), the general public's understanding of their use is very limited. When people have information about the presence of a nanomaterial in a product, most (62%) consider them as not as safe as products not containing NMs, avoid it, and determine themselves based on the product category. People are more suspicious of food, health products, and cosmetics. However, they are more confident (half of them) in NM-containing health products than in products of other categories. Overall, consumers' perception of risk is highly dependent on the applications of the products concerned. As a result, exposure to NMs in sunscreens, drugs or cosmetics is considered riskier by the public than in electronic products (zero or lower exposure). For example, two-thirds of respondents are concerned about direct contact with NMs. It should also be noted that 87% of respondents considered that they should know when they buy a product containing a nanomaterial.

Patient organizations are alerting the European Commission and have made recommendations for better information on applications, benefits and risks and greater traceability about NMs in health products (39, 40). Their demands also concern the protection of the environment and health.

Thus, understanding the use of nanoparticle forms in health products is very complex for the public. This is easily understandable due to the wide variety of products and applications but also because of different definitions and regulations depending on the product. The fact that traceability and systematic labeling are not mandatory can contribute to mistrust. Nanotechnology debates and concerns in societies exemplify as well the relationship that Americans and Europeans have with such innovations and provides a relevant focus for a renewed analysis of democracy (41).

## Environmental topics

Regarding ecotoxicological effects, research has highlighted potential risks of NMs to invertebrates and fish, including effects on behavior, reproduction and development (27, 42). Thus, NMs -health products are likely to affect the different physical (atmosphere, water, soil, sediment) or biological compartments of the environment.

The study of the fate and behavior of NMs in health products, such as their interaction with different compartments, including living organisms, their bioavailability, bioaccumulation, biodegradation as well as the modification of their physicochemical properties is an important task to deal with. This approach is currently, as for any drug, carried out by applying the usual tests recommended in the document “*Guideline on the environmental risk assessment of medicinal products for human use” EMEA/CHMP/SWP/4447/00* (43) on the assessment of the environmental risk of medicinal products based on a control of the physicochemical properties of nanomedicines.

Research on the impact of eliminating drugs and their degradation products, including possible NMs, in the environment, was considered insufficient (44). Most of the studies and work carried out to date concern the risks of NMs in food, following occupational exposures and in the environment. As such, in France, Agence Nationale Sécurité Sanitaire Alimentaire Nationale (ANSES) and Institut national de l'environnement industriel et des risques (INERIS) have carried out a great deal of work, relying in particular on the use of data from the R-nano register (45), which compiles the mandatory declarations of these products.

## Actionable recommendations and perspective

The current burst of technologies and possibilities in the nanotechnology field is close to the one which prevailed in previous years with classical chemistry and, more recently, with some biologicals. Processes and regulations must therefore adapt to these new paradigms. In this respect, four main areas were identified.

i) The first one relates to accessibility to clinical research. Because of their specific properties, NMs are challenging for their characterization and mass production. Defining regulatory standards and critical quality attributes (preferred approach as well as that of quality-by-design), as well as specific non-clinical tests seems of paramount importance. In the longer term, this will make it possible to harmonize in successive stages the international regulations applicable to NMs in health products. It would therefore as well be interesting to recommend the implementation of a European workshop, like the one that took place about 10 years ago (46), to study the possible need for new recommendations.

Work should also be done to harmonize regulations at the international level to facilitate patients' access to these health innovations. To do this, it is necessary to mobilize existing ICH working groups on these specific nano-related topics, *via* the EMA, and building on the ongoing work [European Nanomedicine Characterization Laboratory (EU NCL), US-NCL, Joint Research Center (JRC), International Pharmaceutical Regulators Programme Nanomedicine Working Group (IPRP)].

ii) The second main identified area relates to risk-benefit balance determination and clinical research. It encompasses a wider field that includes also traditional excipients (authorized in Europe as food additive) that contain NMs, and other nanoparticle-concerned products. A risk assessment methodology is proposed (Table 1).

**Table 1:** Actionable recommendations, actors, and possible actions.

**Accessibility to clinical research**	**Actors**	**Possible actions**
• Promote and stimulate research and development on new methods and techniques for assessing the quality and safety of nanomedicines: - Define regulatory standards on expected characterizations and critical quality attributes and critical process parameters - Develop, identify and standardize relevant and adapted characterization methods (metrology, assay) - Stimulate research in the specific study of the carcinogenicity, reprotoxicity and immunotoxicity of NMs	Applied research and regulatory science	=> Raise the subject of the establishment of networks of European research laboratories: JRC, European Commission => Initiate with the EMA a new European workshop on NMs and medicines
• Harmonize international regulations on NMs for nanomedicines	European (EMA) and international regulatory agencies, ICH groups, industrials.	=>Port this topic to the EMA, and to international groups (ICH)
• Develop relevant approaches on the equivalence and interchangeability of nanosimilars	Researchers and regulatory agencies	=>Research; calls for tenders for methods for determining equivalence =>Regulation, see possibilities depending on what has been done for biosimilars (see JRC Refine)
**Benefit/risk analysis and surveillance**	**Actors**	**Possible actions**
• Define and develop an unambiguous definition of NMs in health products (in terms of size, proportion, and possibly use), to ensure traceability (evaluation, pharmacovigilance, pharmacoepidemiology)	European Commission, EMA and international regulatory agencies, ICH groups	=>International harmonized definition (*via* ICH groups for example) of nanodrugs and NMs in health products: EMA
• Improve traceability (reporting) of some health products (excipients and MDs) containing NMs	International and European levels: regulatory agencies and industrials	=> Bring this topic (mandatory declaration and labeling) to the European Commission (medicines and MDs) and international health policy bodies
• Precisely define and articulate the respective roles of pharmacovigilance and pharmacoepidemiology studies and define tools (including databases and their access) to better detect possible remote (delayed) effects of health products containing NMs	At regulatory agencies and research laboratories	=> For MDs, to be considered also with regard to unintentional releases (epidemiology)
• Establish systems to quantify and assess the nature and consequences of nanoparticle releases (wear) by MDs	Researchers	=>Calls for tenders: strong incentives to create new models to study the potential effects of these releases.
• Revisit the benefit-risk balance of the NMs included in the excipients, requiring improved traceability (proportion of nanomaterial, exact nature, etc.), and considering all their roles, including in the stability of the active ingredients and in the compliance of treatments (e.g., opacity, color)	At European (EMA) and international regulatory agencies, ICH groups	=> Bring this topic to the regulatory agencies: Subject to deepen and study for several excipients => Bring this topic to the regulatory agencies: Changing regulations (traceability, reporting,…)
**Societal and environmental topics**	**Actors**	**Possible actions**
• Develop methods and systems to determine the fate of NMs from health products in the environment	Researchers and agencies	Research actions (preferably with agency and laboratory interfaces): => Dedicated calls for tenders. To be studied in the same context as that of pollution by drugs, but dedicated methods potentially necessary (see characterization above, from which also derives research needs)
• Improve the general public's knowledge of NMs in health products (in particular on the risk-benefit balance): communication and transparency	Health authorities, departments, industrials	=>Communication campaigns on the role and nature of nanoscale =>Labeling: transparency, public information

Main recommendations (actions) by topics (accessibility to clinical research, benefit-risk analysis and surveillance, societal and environmental topics), with potential actors.

When it comes to carrying out surveillance studies, ensuring full traceability of the nanomaterial in the health product for retrospective epidemiological studies seems essential. Indeed, currently, it is quite possible to put on the market drugs containing nanoparticle forms without the exact quantity of NMs being known or traceable (case of excipients in conventional chemical drugs). The case of by-products resulting from MDs wear is also another potentially important topic.

For several stakeholders involved (mainly clinical researchers, industrials and regulators), achieving a consensus and international reference definition of NMs in health products (at least by product type) would be important and require further work. This would allow an informed evaluation by the authorities and a full follow-up of the clinical trial to pharmacovigilance (and pharmacoepidemiology).

iii) Improving knowledge about the nature, quantity, proportion, and documented benefit-risk balance of NMs in health products, beyond allowing for more relevant assessments and monitoring, would also greatly contribute public debates. Improving transparency and health democracy [systematic involvement of patient associations and representative in health policy-defining working groups (47)] on ongoing or planned work should therefore help in this area (Table 1).

iv) Beyond the questions relating to the acceptability of the risk of health products related to NMs for the treated individual, there are also those related to the possible release of NMs into the environment (Table 1).

Many actions and works have already been implemented (such as with the EU NCL) or are in progress. Indeed, the EMA has been recognizing the specific challenges posed by nanomedicines since 2009. Thus, the CHMP (The Committee for Medicinal Products for Human Use) of the EMA organized a symposium dedicated to nanomedicines, followed by an international workshop and various Reflection papers (23, 46, 48, 49). Since 2014, it has also set up an Innovation Task Force that brings together the multidisciplinary scientific and regulatory expertise of member states' agencies, to help implement emerging therapies such as nanomedicines. In 2017, the JRC, under the leadership of the European Commission, launched the REFINE project. The JRC recently published a White Paper with the aim of anticipating regulatory needs for innovative products using nanotechnologies (14). This work was the result of the 2019 Global Regulatory Science Summit (GSRS19) co-hosted by the JRC and the Global Coalition for Regulatory Scientific Research (GCRSR), with experts from around the world. On 31 March 2020, the EMA published its regulatory strategy for the period 2020–2025 (EMA strategy) including nanotechnologies and innovative products in health products. The International Pharmaceutical Regulators Program has also a dedicated nanomedicine working group which works on the exchange of information between regulators.

## Conclusion

The multidisciplinary approach chosen for this work was an opportunity to highlight the various scientific and societal challenges posed by NMs in health products to regulators.

Concerted and cross-discipline efforts have to be pursued to reach common grounds of understanding, which may then help adopting common rules. These efforts should be global in the sense of worldwide work but also in including all stakeholders, such as patient associations and non-governmental organizations. They can come from academic or private research and regulation evolutions. Regulatory agencies can also stimulate some of these innovations through a requirement for results at the level of regulatory texts, but the bulk of the efforts must be funded and stimulated by the authorities supervising research at European or national levels. Indeed, further scientific research is needed to provide sound scientific bases for an adequate assessment of the quality, safety and efficacy of emerging nanotechnologies used in health products. It is logical and desirable to continue to promote the emergence of networks of laboratories (with peer review, exchange of reference materials, blind tests, etc.) in these fields. In this respect, the following areas for improvement were identified.

- For nanomedicines, international approximation and harmonization of quality regulatory standards and non-clinical assays, when existing standards do not apply, would greatly improve accessibility to nano-enabled innovative drugs.
- Improving scientifically-based risk-benefit balance determination (for nanomedicines but also for traditional excipients and medical devices) to be used for marketing authorization but also for post-market surveillance is essential. This would require traceability and unambiguous international reference definitions.
- Proactively considering general public perception of nano-sized materials in health products and of their potential consequences to the environment would also contribute positively to on-going public debates in the field.

This global analysis also showed that health democracy and transparency toward the general public should be strengthened. Regulatory decisions and scientific evidence are not enough to gain acceptance and trust. Involving patients in this harmonization and providing them with clear and transparent information about these products will make it easier for the general public to understand the benefits or risks of such innovations.

## List of the members of the Scientific Council of ANSM

Joël Ankri (Chairman), Janine Barbot, Robert Barouki, Henri Bastos, Éric Bellissant, Christiane Druml, Éric Ezan, Didier Houssin, Walter Janssens, Marie-Christine Jaulent, Maria Emilia Monteiro, Dominique Pougheon, Vololona Rabeharisoa, Victoria Rollason, Valérie Sautou, Jean-Paul Vernant.

## Author contributions

WO-G led the nanomaterials working group and wrote the manuscript. JA chaired the Scientific Council and working group and supervised the work with PM. VS, EE, HB, EB, and LB contributed their expertise during ANSM working group sessions and manuscript design and revisions. All authors reviewed the manuscript and provided feedback and edits. All authors approved the final manuscript and the decision to submit for publication.
